# Markedly Melanin-Pigmented Paraganglioma Associated With Decreased Dopa-Decarboxylase Expression

**DOI:** 10.1210/jcemcr/luaf208

**Published:** 2025-10-07

**Authors:** Maho Taguchi, Junichiro Sato, Yoichi Yasunaga, Maki Takeuchi, Tetsuo Ushiku, Noriko Makita

**Affiliations:** Department of Nephrology and Endocrinology, Graduate School of Medicine, The University of Tokyo, Tokyo 113-8655, Japan; Department of Nephrology and Endocrinology, Graduate School of Medicine, The University of Tokyo, Tokyo 113-8655, Japan; Department of Pathology, Graduate School of Medicine, The University of Tokyo, Tokyo 113-8655, Japan; Department of Nephrology and Endocrinology, Graduate School of Medicine, The University of Tokyo, Tokyo 113-8655, Japan; Department of Pathology, Graduate School of Medicine, The University of Tokyo, Tokyo 113-8655, Japan; Department of Nephrology and Endocrinology, Graduate School of Medicine, The University of Tokyo, Tokyo 113-8655, Japan

**Keywords:** pigmented tumor, pheochromocytoma, paraganglioma, dopamine, L-DOPA

## Abstract

A 49-year-old woman undergoing evaluation for sigmoid colon cancer was incidentally found to have a 56-mm retroperitoneal tumor. Although asymptomatic, her blood dopamine level was elevated to 783 pg/mL (reference range, ≤20 pg/mL [SI: ≤79.55 pmol/L]). Magnetic resonance imaging revealed high intensity on T2-weighted and diffuse high intensity on fat-suppressed T1-weighted images, suggestive of a melanin-producing paraganglioma (PGL). The surgically resected tumor appeared black macroscopically, and histological examination showed brown melanin granules on hematoxylin and eosin staining, consistent with pigmented PGL. This rare tumor's pathogenesis remains poorly understood. Catecholamines and melanin both originate from tyrosine via L-DOPA. Normally, in the adrenal medulla and ganglia, L-DOPA is converted to dopamine by dopa decarboxylase (DDC). However, decreased DDC activity can lead to elevated L-DOPA levels, which may contribute to melanin synthesis and pigmentation. Since L-DOPA secreted into the blood is rapidly converted to dopamine by vascular endothelial DDC, blood dopamine can rise even without a dopamine-producing tumor. In this case, DDC immunostaining was negative, suggesting an L-DOPA-producing tumor. Based on these findings, we hypothesize that all pigmented PGLs may represent L-DOPA–producing tumors.

## Introduction

Pheochromocytoma/Paraganglioma (PPGL) is a type of tumor that produces catecholamines and develops from chromaffin cells located in either the adrenal medulla or the paraganglia [1]. Tumors that originate in the adrenal medulla are called pheochromocytomas (PCC), whereas those that arise in the paraganglia are referred to as paragangliomas (PGL) [1]. PPGLs typically exhibit elevated levels of epinephrine and norepinephrine; however, there are rare cases in which the tumors exhibit elevated dopamine levels [2, 3]. Many PPGLs with elevated dopamine levels, considered dopamine-producing tumors, are thought to be poorly differentiated and potentially metastatic [4]. Due to the rarity of these tumors, our understanding of the dynamics of catecholamine-synthesizing enzymes within the tumors is still limited. Additionally, some PPGLs may rarely cause pigmentation, possibly due to melanin deposition, though the mechanism remains unclear [5]. We recently experienced a case of PGL with high plasma dopamine levels and pigmentation. Based on the immunohistochemical analysis of this case, we suggest that the pigmented PGL might be associated with an intratumoral enzymatic pathway that leads to melanin production due to the overproduction of L-DOPA instead of dopamine.

This is the first report to describe both the imaging diagnosis and immunohistopathological findings of pigmented PGL.

## Case Presentation

**Figure 1.**
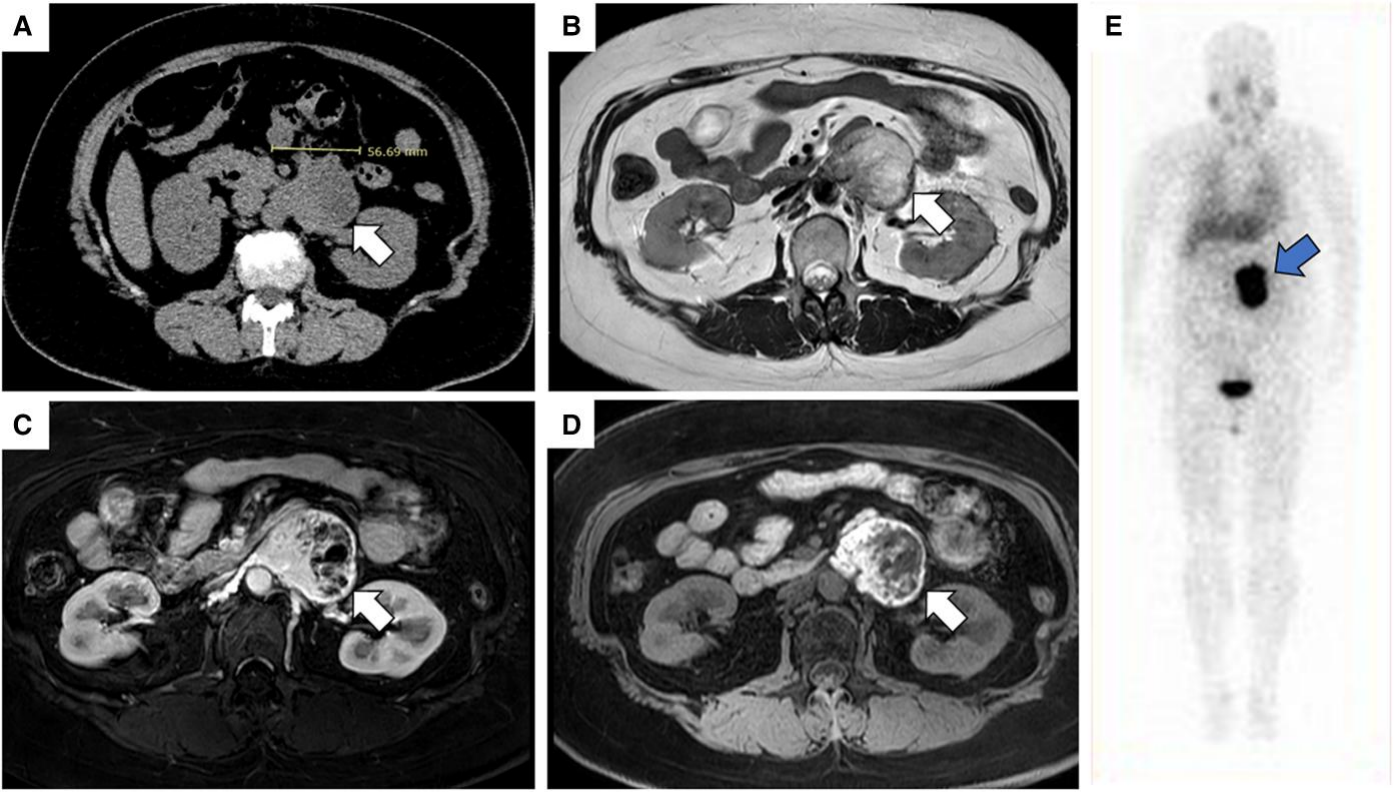
Images displaying a retroperitoneal tumor. A, Plain computed tomography revealed a 56-mm tumor. B, T2-weighted magnetic resonance imaging (MRI) showed a tumor with high intensity. C, Contrast-enhanced MRI showed contrast enhancement in the early phase. D, Fat-suppressed T1-weighted MRI showed a diffuse high intensity. E, Iodine-123 metaiodobenzylguanidine (¹²³I-MIBG) scintigraphy demonstrated an intense uptake consistent with the tumor.

A 49-year-old woman was referred to our department because a 56-mm retroperitoneal tumor was incidentally discovered on a plain computed tomography (CT) scan for close examination of early-stage sigmoid colon cancer (Fig. 1A). She had no symptoms of catecholamine excess such as hypertension, headache, palpitations, or sweating.

She had diabetes and was taking oral hypoglycemic agents (sitagliptin and metformin). Her past medical history included a unilateral adnexectomy performed 10 years ago for a chocolate cyst. Her family history revealed that both parents had hypertension and diabetes, but there was no family history of PPGL, neurofibromatosis type 1, von Hippel-Lindau disease, or multiple endocrine neoplasia.

## Diagnostic Assessment

The patient had a high blood dopamine level of 783 pg/mL (reference range, ≤20 pg/mL [SI: ≤79.55 pmol/L]). Only urinary normetanephrine was slightly above the reference range (0.51 mg/day, reference range, 0.09-0.33 mg/day [SI:4.91-18.0 µmol/L]), but plasma epinephrine/norepinephrine levels and metanephrine/normetanephrine levels were both within the normal range. Table 1 shows the background information and admission laboratories. The T2-weighted magnetic resonance image (MRI) showed a high intensity, and contrast-enhanced MRI showed contrast enhancement in the early phase, leading to a suspicion of PGL (Fig. 1B and 1C). Additionally, the fat-suppressed T1-weighted MRI showed a diffuse high signal (Fig. 1D). This led to suspicions of high protein content, hemorrhage, or melanin. However, the presence of contrast enhancement in the mass made the possibility of high protein content less likely, and the diffusibility of the finding did not strongly suggest hemorrhage, leading to the conclusion that melanin was the most likely component. Iodine-123 metaiodobenzylguanidine (¹²³I-MIBG) scintigraphy revealed abnormal accumulation corresponding to the retroperitoneal mass, which was considered consistent with PGL (Fig. 1E). Based on the aforementioned findings, pigmented PGL caused by melanin deposition was suspected.

**Table 1.** Patient and laboratory data characteristics on admission

Clinical characteristics		
Age, y	49	
Sex	Female	
Height	151 cm	
Weight	69 kg	
Body mass index	30.2	
Blood pressure	115/83 mm Hg	
Heart rate	90 bpm	
Respiratory rate	17/min	
Body temperature	35.4 °C	
**Laboratory data**		Reference range
WBC	8.80 × 10^3^/μL	3.5-9.0 × 10³/μL
	(8.80 × 10⁹/L)	(3.5-9.0 × 10⁹/L)
Neutro	67.50%	40%-70%
Lym	25.90%	20%-45%
Eosino	2.30%	0%-6%
Hemoglobin	15.6 g/dL	11.5-15.0 g/dL
	(156 g/L)	(115-150 g/L)
Albumin	4.6 g/dL	3.9-4.9 g/dL
	(46 g/L)	(39-49 g/L)
AST	32 U/L	13-30 U/L
	(0.53 μkat/L)	(0.22-0.50 μkat/L)
ALT	55 U/L	10-40 U/L
	(0.92 μkat/L)	(0.17-0.67 μkat/L)
Total bilirubin	0.4 mg/dL	0.2-1.2 mg/dL
	(6.84 μmol/L)	(3.4-20.5 μmol/L)
Creatinine	0.49 mg/dL	0.6-1.1 mg/dL
	(43.32 μmol/L)	(53-97 μmol/L)
eGFR	102.4 mL/min/1.73 m^2^	>60 mL/min/1.73 m^2^
C-reactive protein	0.08 mg/dL	<0.3 mg/dL
	(0.8 mg/L)	(<3 mg/L)
Glucose	78 mmol/L	70-99 mmol/L
	(4.33 mmol/L)	(3.9-5.5 mmol/L)
HbA_1c_	7.00%	4.6%-6.2%
	(53 mmol/mol)	(27-44 mmol/mol)
Epinephrine	47 pg/mL	<100 pg/mL
	(0.26 nmol/L)	(<0.55 nmol/L)
Norepinephrine	439 pg/mL	100-450 pg/mL
	(2.59 nmol/L)	(0.591-2.660 nmol/L)
Dopamine	783 pg/mL	<20 pg/mL
	(4102.92 pmol/L)	(<79.55 pmol/L)
Metanephrine	42.6 pg/mL	<130 pg/mL
	(215.98 pmol/L)	(<659 pmol/L)
Normetanephrine	246.1 pg/mL	<506 pg/mL
	(1343.71 pmol/L)	(<2762 pmol/L)
ACTH	28.51 pg/mL	7.2-63.3 pg/mL
	(6.28 pmol/L)	(1.6-13.9 pmol/L)
Cortisol	7.9 µg/dL	4.0-18.3 µg/dL
	(217.96 nmol/L)	(110-504 nmol/L)
DHEA-S	38 µg/dL	19-231 µg/dL
	(1.03 μmol/L)	(0.52-6.27 μmol/L)
Aldosterone	32.3 pg/mL	30-160 pg/mL
	(89.47 pmol/L)	(83-443 pmol/L)
Renin activity	0.8 ng/mL/h	0.3-2.9 ng/mL/h
Urinary metanephrine	0.301 mg/gCr	0.04-0.19 mg/gCr
	(1.53 μmol/mol・Cr)	(0.20-0.96 μmol/mol・Cr)
Urinary normetanephrine	0.113 mg/gCr	0.09-0.33 mg/gCr
	(0.62 μmol/mol・Cr)	(0.49-1.80 μmol/mol・Cr)
Urinary metanephrine	0.13 mg/d	0.04-0.19 mg/d
(Urine 1800 mL/d)	(0.66 μmol/d)	(0.20-0.96 μmol/d)
Urinary normetanephrine	0.51 mg/d	0.09-0.33 mg/d
(Urine 1800 mL/d)	(2.78 μmol/d)	(0.49-1.80 μmol/d)

Values in parenthesis are International System of Units (SI).

Abbreviations: ACTH, adrenocorticotropin; ALT, alanine transaminase; AST, aspartate transaminase; DHEA-S, dehydroepiandrosterone sulfate; eGFR, estimated glomerular filtration rate; Eosino, eosinophil; HbA_1c_, glycated hemoglobin A_1c_; Lym, lymphocytes; Neutro, neutrophil; WBC, white blood cell.

## Treatment

Although the tumor appeared dopamine dominant, mechanical manipulation during surgery could still release epinephrine or norepinephrine and trigger a hypertensive crisis. Therefore, the patient was admitted for preoperative α-blockade. With a doxazosin dose of 10 mg/day, saline dose of 1000 mL/day, and salt dose of 3 g/day, her blood pressure stabilized. One week later, she underwent retroperitoneal tumor resection in the urology department. Blood pressure remained stable postoperatively, and she was discharged 2 weeks later with good recovery.

**Figure 2.**
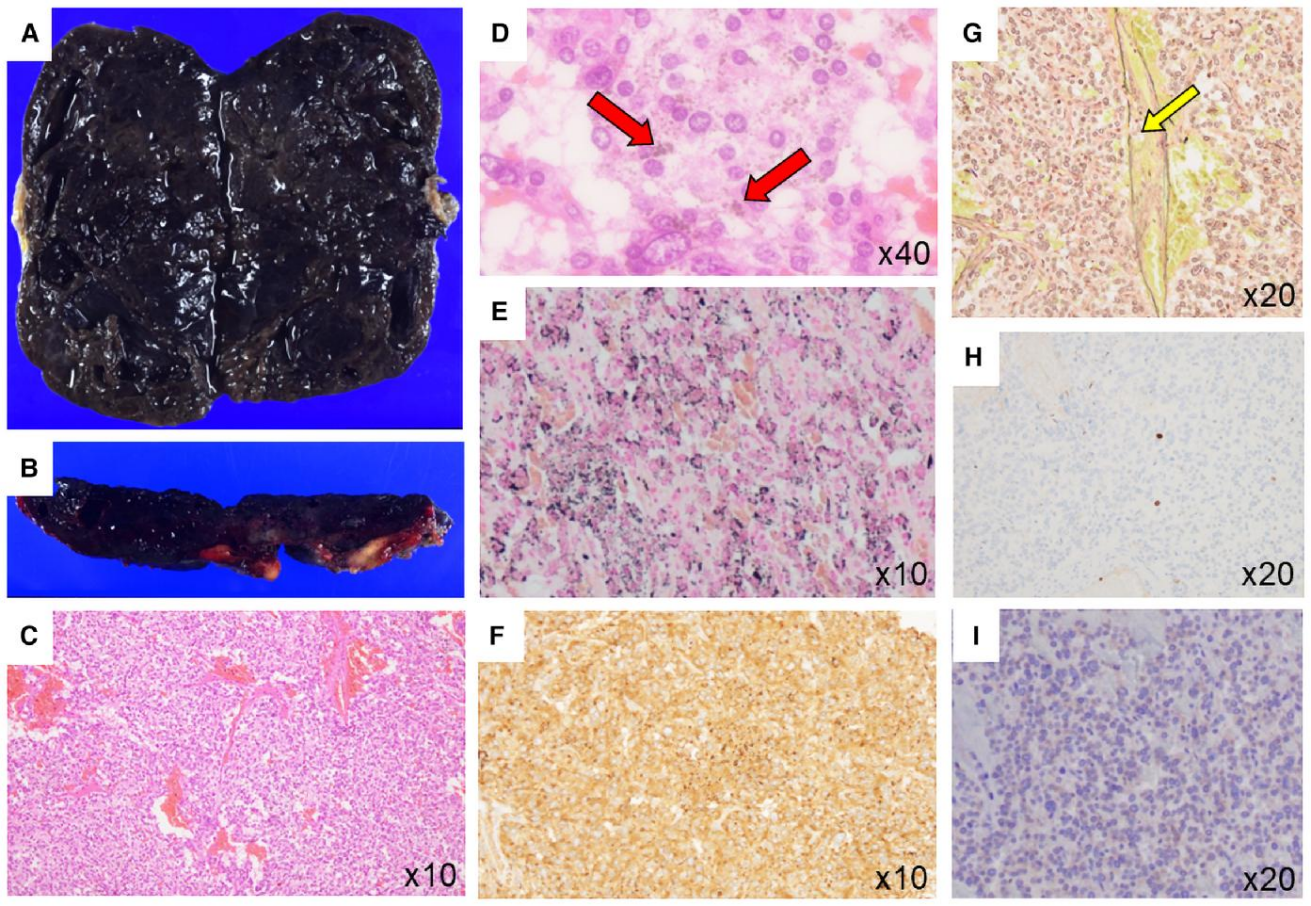
A and B, Macroscopic appearance and C to G, histopathological images of the resected tumor. A and B, Grossly, the cut surface of the tumor exhibited a tar-like black appearance. C and D, Hematoxylin and eosin (HE) stain. C, At low power magnification, the tumor was characterized by large and irregular cell nests with high cellularity. D, At high-power magnification, the neoplastic cells contained melanin pigments, indicated by arrows. E, Melanin pigments are positive for Fontana-Masson stain. F, Immunohistochemically, the neoplastic cells were positive for chromogranin A. G, Elastica van Gieson stain showed vascular invasion, indicated by an arrow. H, Immunohistochemically, Ki-67 labeling index was less than 1%. I, Immunohistochemically, the neoplastic cell were positive for succinate dehydrogenase complex subunit B (SDHB).

The cut surface of the resected tumor exhibited a tar-like black appearance (Fig. 2A and 2B). Hematoxylin and eosin (HE) staining demonstrated a Zellballen pattern composed of neoplastic cells containing melanin pigments (Fig. 2C and 2D). Melanin deposition was also confirmed by Fontana-Masson staining, which showed punctate black pigmentation (Fig. 2E).

**Figure 3.**
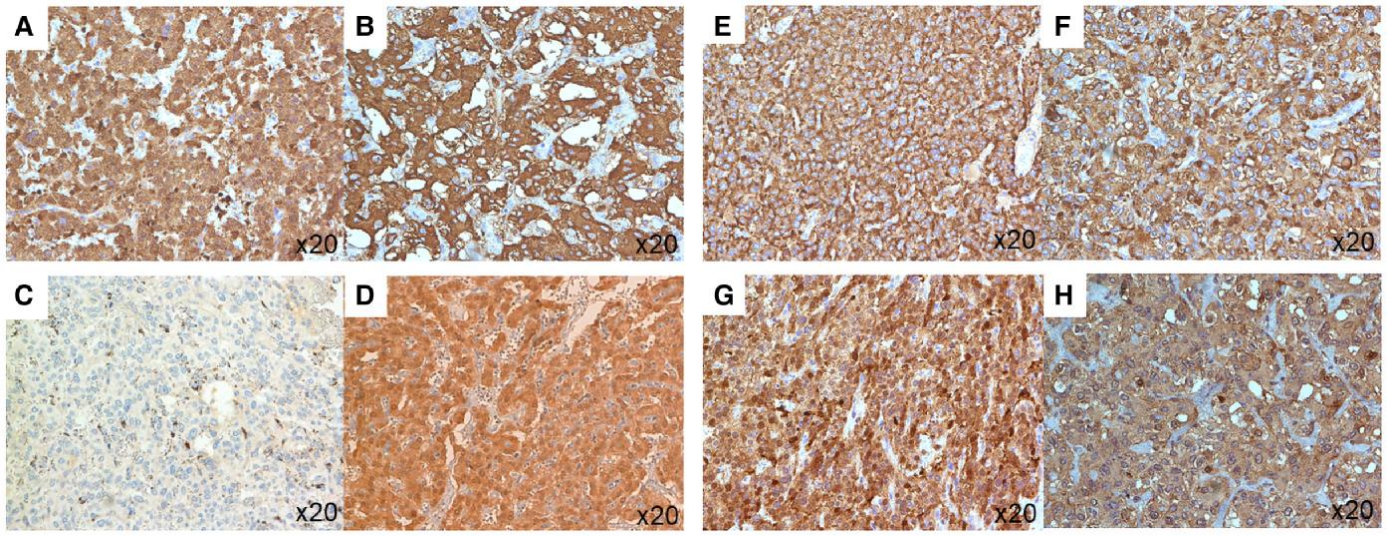
An immunohistological analysis of catecholamine synthesis enzymes. Tyrosine hydroxylase (TH), which converts tyrosine to L-DOPA; dopamine β-hydroxylase (DBH), which converts dopamine to norepinephrine; and phenylethanolamine *N*-methyltransferase (PNMT). A, The neoplastic cells of our present case were positive for TH. B, The neoplastic cells of the control case were positive for TH. C, The neoplastic cells of our present case were negative for dopa decarboxylase (DDC). D, The neoplastic cells of the control case were positive for DDC. E, The neoplastic cells of our present case were positive for dopamine β-hydroxylase (DBH). F, The neoplastic cells of the control case were positive for DBH. G, The neoplastic cells of our present case were positive for phenylethanolamine N-methyltransferase (PNMT). H, The neoplastic cells of the control case were positive for PNMT.

Immunohistochemical analysis revealed that the tumor cells were positive for chromogranin A (Fig. 2F), with scattered S100-positive sustentacular cells present. Based on these findings, a diagnosis of pigmented PPGL was established. The tumor was characterized by large, irregular cell nests with high cellularity and the presence of vascular invasion (Fig. 2G). Ki-67 labeling index was less than 1% (Fig. 2H). Consequently, the GAPP (Grading, Activity, Proliferation, and Pheochromocytoma/Paraganglioma) score, which serves as an indicator of PPGL malignancy [6], was determined to be 4, indicating moderate malignancy. Immunohistochemically, succinate dehydrogenase complex subunit B (SDHB) was positive (Fig. 2I). Expression of intratumoral catecholamine synthases was evaluated by immunohistochemistry for the present case (Fig. 3A, 3C, 3E, and 3G) and the control case of paraganglioma with normal dopamine levels (Fig. 3B, 3D, 3F, and 3H). Tyrosine hydroxylase (TH), which converts tyrosine to L-DOPA; dopamine β-hydroxylase (DBH), which converts dopamine to norepinephrine; and phenylethanolamine *N*-methyltransferase (PNMT), which converts norepinephrine to epinephrine, were positive in both cases. However, dopa decarboxylase (DDC), the enzyme responsible for converting L-DOPA to dopamine, was negative in the present case in contrast to the control case.

## Outcome and Follow-up

One month after the operation, the patient’s serum dopamine level had dropped to 18 pg/mL. After that, blood tests and CT scans have been performed every 3 months at the urology outpatient clinic. The patient has been followed up without any recurrence.

## Discussion

We present a rare case of pigmented PGL. In vivo, pigmentation can involve substances such as melanin, hemosiderin, lipofuscin, and chromaffin. Pigmented tumors include malignant melanoma, schwannoma, ganglioneuroblastoma, pulmonary carcinoid tumor, medullary thyroid carcinoma, dermatofibrosarcoma, and adrenal cortical adenoma [7].

Pigmented PGL is extremely rare, with only 30 cases reported, and many of its features remain unclear [8]. Imaging evaluations such as CT or MRI were lacking in several cases. In our case, high signal intensity on T1-weighted imaging led to the preoperative suspicion of a melanin-pigmented tumor. Melanin-containing tumors like melanoma often show high T1 signal [9]. Pathological features include macroscopic black pigmentation, brownish granules on HE or Fontana-Masson staining, and pigment granules seen on electron microscopy [10, 11]. Catecholamine measurements vary; only one case showed elevated dopamine levels, similar to ours [8]. Tumors are typically larger than 4 cm, with no metastasis at diagnosis. Notably, all reported cases were in female patients [8]. These features align with our case.

**Figure 4.**
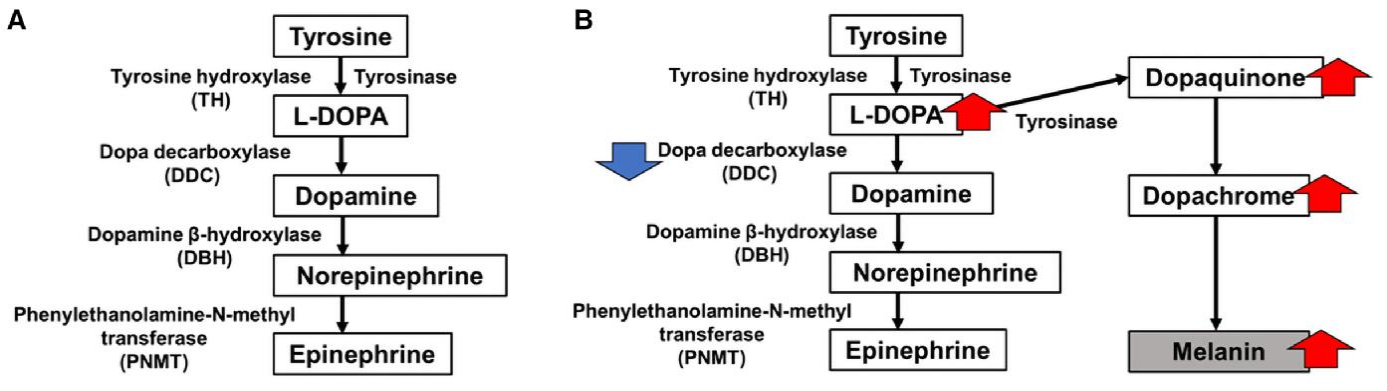
Catecholamines and melanin synthesis pathway. A, Catecholamines are produced from tyrosine via L-DOPA, dopamine, norepinephrine, and epinephrine. B, In our present case, the suppression of dopa decarboxylase prevents L-DOPA from being converted into dopamine. Consequently, L-DOPA is overproduced while dopamine is not. Some of the excess L-DOPA contributes to melanin synthesis via an alternative pathway.

**Figure 5.**
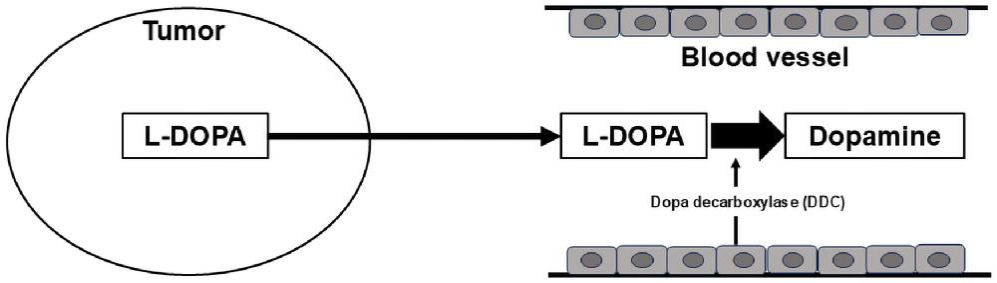
The mechanism by which L-DOPA–producing tumors cause high blood levels of dopamine. When L-DOPA is overproduced in a tumor, it flows into the bloodstream and is quickly converted into dopamine by dopa decarboxylase in the vascular endothelial cells. Therefore, laboratory tests also revealed high blood dopamine levels, similar to the conventional exclusive dopamine-producing pheochromocytoma/paraganglioma tumor.

Pigmented PPGL has traditionally been attributed to abnormal differentiation of adrenal medullary cells (neural crest origin) or to degradation products of intratumoral catecholamines [5]. More recently, another mechanism was proposed [8]. Catecholamines are synthesized via conversion of tyrosine to L-DOPA by TH, then to dopamine by DDC, norepinephrine by DBH, and epinephrine by PNMT (Fig. 4A). Melanin, in contrast, is synthesized when tyrosine is converted to L-DOPA and then to dopaquinone by tyrosinase, eventually forming melanin. Increased L-DOPA enhances melanin production. Normally, L-DOPA is rapidly converted to dopamine by DDC in the adrenal medulla and sympathetic ganglia. However, reduced DDC activity may elevate L-DOPA levels, promoting pigmentation via melanin biosynthesis (Fig. 4B). L-DOPA is also rapidly converted to dopamine in the blood by DBH, potentially explaining elevated dopamine even in non–dopamine-producing tumors (Fig. 5). Although DBH activity is said to be reduced in dopamine-producing tumors [4], DBH staining was positive in this case, suggesting otherwise. According to this hypothesis, melanin would not be produced without increased L-DOPA. In our case, immunostaining for DDC was negative, suggesting that this tumor may have produced L-DOPA. Previously reported pigmented PPGLs may also have been L-DOPA–producing tumors.

Nonetheless, we acknowledge that the co-occurrence of pigmentation and decreased DDC expression, while mechanistically plausible, may be coincidental. Given the extreme rarity of pigmented PPGLs and limited reports with detailed biochemical or immunohistochemical evaluation, a causal relationship remains speculative.

To investigate the mechanism of DDC suppression, we performed whole-exome sequencing, but found no pathogenic variants in the *DDC* gene, including exon-intron boundaries. Possible mechanisms include epigenetic silencing through promoter methylation or histone modification, mutations in enhancers or repressors regulating DDC transcription, aberrant RNA splicing, posttranscriptional regulation by microRNAs, or chromosomal structural abnormalities.

DBH activity may be reduced in undifferentiated tumors despite preserved expression [12]. Although electron microscopy was not performed, several findings suggest preserved differentiation in this case. The tumor showed clear ^123^I-MIBG uptake, indicating intact volumetric modulated arc therapy function, and histologically displayed typical paraganglioma features, including chromogranin A positivity and low proliferative activity.

These findings support the notion that the decreased DDC expression was not due to pathogenic variants or cellular immaturity, but more likely due to transcriptional or posttranscriptional dysregulation.

Regarding the prognosis of pigmented PPGLs, malignant behavior has been reported only rarely among 30 cases described to date. In the present case, the GAPP score was 4, indicating moderate malignancy; however, the Ki-67 index was low and no metastasis was observed. In contrast, dopamine-producing PPGLs are generally considered highly malignant. If pigmentation in pigmented PPGLs is indeed due to DDC downregulation, then decreased DDC expression does not necessarily indicate undifferentiation or malignancy. Further case accumulation is essential to clarify the prognosis of pigmented PPGLs.

The limitation of this case report is that it describes only a single case. Further accumulation of similar cases is needed in the future.

This is the first case report of a pigmented PGL in which both imaging and pathologic findings are consistent. There are few previously reported cases of pigmented PPGL, so further case series are warranted to clarify the characteristics of pigmented PPGL.

## Learning Points

- When DDC activity is decreased, L-DOPA increases because L-DOPA is not converted to dopamine; L-DOPA is converted to dopaquinone by tyrosinase, and melanin is ultimately produced from dopaquinone, which may result in pigmentation.
- Since DDCs are also present in vascular endothelial cells, L-DOPA is quickly converted to dopamine in the blood. Therefore, L-DOPA–producing tumors have high levels of dopamine in the blood even in the absence of excessive dopamine production from the tumor.
- Pigmented PPGLs might be L-DOPA–producing tumors.

## Data Availability

Original data generated and analyzed during this study are included in this published article.

## Abbreviations

¹²³I-MIBG: iodine-123 metaiodobenzylguanidine
CT: computed tomography
DBH: dopamine β-hydroxylase
DDC: dopa decarboxylase
GAPP: Grading, Activity, Proliferation, and Pheochromocytoma/Paraganglioma
HE: hematoxylin and eosin
MRI: magnetic resonance imaging
PGL: paraganglioma
PNMT: phenylethanolamine *N*-methyltransferase
PPGL: pheochromocytoma/paraganglioma
SDHB: succinate dehydrogenase complex subunit B
TH: tyrosine hydroxylase
